# The STAP-study: The (cost) effectiveness of custom made orthotic insoles in the treatment for plantar fasciopathy in general practice and sports medicine: design of a randomized controlled trial

**DOI:** 10.1186/s12891-016-0889-y

**Published:** 2016-01-16

**Authors:** N. Rasenberg, L. Fuit, E. Poppe, A. J. A. Kruijsen-Terpstra, K. J. Gorter, M. S. Rathleff, P. L. J. van Veldhoven, P. J. Bindels, S. M. Bierma-Zeinstra, M. van Middelkoop

**Affiliations:** Department of General Practice, Erasmus MC, University Medical Centre, Rotterdam, PO Box 2040, 3000 CA Rotterdam, The Netherlands; Podotherapie Fuit, Schaapweg 10c, 2285 SP Rijswijk, The Netherlands; Podotherapie Voet op Maat, Kortekade 14A, 3062 GR Rotterdam, The Netherlands; Dutch Association of Podiatrists, Nederlandse Vereniging van Podotherapeuten, Noordse Bosje 18, 1211 BG Hilversum, The Netherlands; Adelbrechtgaarde 5, 7329 AT Apeldoorn, The Netherlands; Research Unit for General Practice in Aalborg, Department of ClinicalMedicine, Aalborg University, DK 9220 Aalborg, Denmark; Department of Sport Medicine, Medical Centre Haaglanden Antoniushove, Leidschendam, PO Box 411, 2260 AK Leidschendam, The Netherlands

**Keywords:** Plantar fasciitis, Plantar fasciopathy, Treatment, Orthotic devices, Podiatrist

## Abstract

**Background:**

Plantar fasciopathy is a common cause of foot pain, accounting for 11 to 15 % of all foot symptoms requiring professional care in adults. Although many patients have complete resolution of symptoms within 12 months, many patients wish to reduce this period as much as possible. Orthotic devices are a frequently applied option of treatment in daily practice, despite a lack of evidence on the effectiveness. Therefore, the objective is to study the (cost)-effectiveness of custom made insoles by a podiatrist, compared to placebo insoles and usual care in patients with plantar fasciopathy in general practice and sports medicine clinics.

**Method/design:**

This study is a multi-center three-armed participant and assessor-blinded randomized controlled trial with 6-months follow-up. Patients with plantar fasciopathy, with a minimum duration of complaints of 2 weeks and aged between 18 and 65, who visit their general practitioner or sport physician are eligible for inclusion. A total of 185 patients will be randomized into three parallel groups. One group will receive usual care by the general practitioner or sports physician alone, one group will be referred to a podiatrist and will receive a custom made insole, and one group will be referred to a podiatrist and will receive a placebo insole. The primary outcome will be the change from baseline to 12 weeks follow-up in pain severity at rest and during activity on a 0–10 numerical rating scale (NRS). Secondary outcomes include foot function (according to the Foot Function Index) at 6, 12 and 26 weeks, recovery (7-point Likert) at 6, 12 and 26 weeks, pain at rest and during activity (NRS) at 6 and 26 weeks and cost-effectiveness of the intervention at 26-weeks. Measurements will take place at baseline and at, 2, 4, 6, 12 and 26 weeks of follow-up.

**Discussion:**

The treatment of plantar fasciopathy is a challenge for health care professionals. Orthotic devices are frequently applied, despite a lack of evidence of the effectiveness on patient reported outcome. The results of this randomized controlled trial will improve the evidence base for treating this troublesome condition in daily practice.

**Trial registration:**

Dutch Trial Registration:NTR5346. Date of registration: August 5^th^ 2015.

## Background

Plantar fasciopathy is a common cause of foot pain in both primary and secondary care. Plantar fasciopathy was formerly known as plantar fasciitis in literature. It typically affects middle aged or older women (40–60 years), with being overweight as a risk factor [1–3]. Besides this, plantar fasciopathy is also commonly seen in highly physically active people; plantar fasciopathy accounts for 8–10 % of all running related injuries [4]. Plantar fasciopathy accounts for approximately 11 to 15 % of all foot symptoms requiring professional care among adults and it has been estimated that a general practitioner (GP), with an average size practice, diagnoses around eight new patients each year [5, 6].

The etiology of plantar fasciopathy is poorly understood and is probably multifactorial [7, 8]. Histopathology studies have indicated that fasciopathy of the plantar fascia might be a possible cause of pain [9, 10]. Repetitive microtrauma and inflammation have been suggested as possible etiologic mechanisms, however these conclusions are mostly based on clinical experience and there is little evidence from research [11–13].

Plantar fasciopathy is characterized by pain over the anteromedial aspect of the inferior heel and the pain tends to increase after periods of inactivity or during weight bearing activities. The diagnosis can be made primarily based on symptoms and physical examination [7]. Patients often report low quality of life and reduced participation as their heel pain prevents them from performing simple every day activities [14, 15]. The clinical course of plantar fasciopathy is considered favorable as 80 % of patients will have a complete resolution of symptoms within 12 months [7]. Despite the favorable clinical course on the long term, many patients do expect and hope for short term pain relief and often multiple treatments are applied during the course of their complaints [7, 16].

The most commonly prescribed treatments include footwear modification, taping, stretching exercises, anti-inflammatory agents, Extra Corporal shock wave therapy (ESWT) and cortisone injections [7, 12]. Reviews conclude that most of these treatments have a role in the management of plantar fasciopathy although at different time points in the clinical course [7, 11–13, 17]. Despite the use of many different treatment strategies in plantar fasciopathy, there is insufficient evidence for the effectiveness of these treatments provided by high quality randomized controlled trials [12, 13, 18, 19].

It has therefore been suggested that treatment options should be offered to the patient in sequence, either based on objective patient criteria or based on the preference of the patient [12]. Different combinations of conservative treatments successfully manage 85–90 % of the cases of plantar fasciopathy [16]. Orthotic devices seem to be a frequently applied treatment option, despite a lack of evidence on the effectiveness [6, 16]. A clinical practice guideline from the American College of Foot and Ankle Surgeons recommends anti-inflammatory drugs (NSAIDs), stretching and prefabricated orthotics as the initial steps in the conservative treatment of plantar heel pain and prescription orthotic devices as a secondary treatment option [6]. Orthotic devices include a low risk intervention, they are relatively painless compared to other treatments, such as shock wave therapy or cortisone injections, and they have a working mechanism that influences on what are thought to be important biomechanical factors in the etiology of plantar fasciopathy. There are different theoretical models on the potential working mechanism of orthotic devices [20]. The most common theory is that orthotic devices optimize the biomechanical loading of the foot, specifically to decrease excessive pronation, to off-load the plantar fascia at its origin and to recreate the heel pad [21].

Orthotic insoles (custom made by a podiatrist) and prefabricated insoles belong to the most researched biomechanical treatments of plantar fasciopathy [12, 18, 19, 22]. However a Cochrane review on the effectiveness of insoles on foot pain concludes that there is insufficient evidence from high quality trials to support the effectiveness of orthotics in patients with plantar fasciopathy on different outcome measures such as pain, function and recovery [22]. Only one study included in this review compared the effectiveness of a custom made insole to a prefabricated insole (non-custom) and a sham insole (placebo) in patients with plantar fasciopathy. Both the custom-made and prefabricated insoles had slightly better outcomes on short term, but were not significantly different and both did not have beneficial effects on pain at 12 months [18].

To our knowledge there is no literature available on the cost-effectiveness of orthotic insoles for the treatment of plantar fasciopathy.

Given the favorability of treatment of plantar fasciopathy with insoles, the high estimated costs involved due to the custom-made nature and the insufficient evidence for this intervention, there is a need to conduct a high quality randomized controlled trial investigating the effectiveness of orthotic insoles on pain, function and recovery in patients with plantar fasciopathy.

### Objectives

The primary objective of our study is to examine the effectiveness of custom made insoles by a podiatrist, compared to placebo insoles and compared to usual care in general practice and sports medicine alone in patients with plantar fasciopathy in terms of pain.

The secondary objective of our study is to examine the cost-effectiveness of custom made insoles by a podiatrist, compared to usual care in patients with plantar fasciopathy.

## Methods

### Study design

**Fig. 1:**
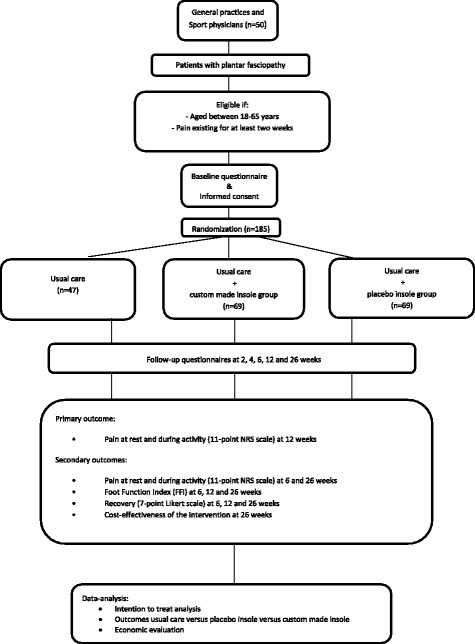
STAP study Flow chart

The STAP-study is a multi-center three-armed armed participant and assessor-blinded randomized controlled trial with 6-months follow-up. STAP is an acronym for: Soles as Treatment Against Pain in feet. The reporting of the study protocol will follow CONSORT guidelines [23]. The study design and flow of participants is shown in Fig. 1. Participants will be recruited in general practice and sports medicine clinics, both accessible without referral. Those who are eligible and give written consent to participate will be randomly assigned to either the usual care group, the placebo insole group and the custom-made insole group. The study is funded by the Netherlands Organization for Health Research and Development (ZonMW) and the Dutch Association of Podiatrists (NVvP). The study design, procedures and informed consent procedure are in compliance with the Declaration of Helsinki, 7^th^ version, October 2013 [24]. The Medical Ethics Committee (number 2015–253) of the Erasmus Medical Centre in Rotterdam has approved this study. This permission includes the multi-centre nature of this study, i.e. approval was given to recruit patients at all sites.

### Patient selection

Patients with plantar fasciopathy, characterized as pain at the medial hind foot, presenting themselves to a GP or sports physician, aged between 18 and 65 years and a minimal pain duration of 2 weeks are eligible for participation.

Participants will be excluded if they have recurrent complaints of plantar fasciopathy for more than 2 years, complaints caused by trauma, earlier treatment for plantar fasciopathy by a podiatrist or treatment with orthotics, suspected (by the GP or sports physician) (osteo)arthritis in the subtalar or talonavicular joint, suspected tarsal tunnel syndrome, suspected stress fractures, infections or tumors in the painful foot or presence of systemic diseases (such as Bechterew’s disease, psoriasis or multiple sclerosis). Patients who have no sufficient understanding of the Dutch language will also be excluded, since they will not be able to complete the questionnaires.

### Sample size

Based on the RCT of Landorf et al. (2006) [18] –pain score 63.4 (SD 21.5), with a difference in pain score of 10.5 (on a scale of 0–100) – it is estimated that with a power of 80 % and an alpha of 0.05 (two-sided), it is possible to detect an effect size of 0.5 with 10.5 points difference on the pain score between patients with a placebo insole and custom made insole with 63 patients in each study group. In addition, a larger difference is expected between the custom made insole group and the usual care group. With a 12-point difference and identical means and SDs, a total of 44 patients are needed per group. A Bonferroni correction will be performed for the secondary comparisons to the usual care group. Taking a loss to follow-up of 10 % into account, a total of 185 patients with plantar fasciopathy (69 in custom made group, 69 in placebo group and 47 in usual care group) will be included in the trial.

### Recruitment of study population

Recruitment of patients will take place in two different settings. All eligible patients who consult a participating GP or sports physician for plantar fasciopathy will be considered for participation (prospective recruitment). On average a GP will see 8 new patients each year. It is estimated that 30 % of these patients will be willing to participate. Therefore, a total of 47 participating GP’s will be necessary to include 185 patients within two years’ time. We aim for a minimum of 50 participating GP’s or sports physicians.

All potentially eligible patients will be invited to participate in the study and will receive comprehensive information about the study. Patients that are willing to participate will fill in a reply card with their contact details and send it to the research team. The research team will then contact the interested patients and inform them further about the study and check the inclusion and exclusion criteria. If a patient is willing to participate in the study and meets all inclusion criteria and none of the exclusion criteria, he or she will sign the informed consent form and send it to the research team. The baseline questionnaire will subsequently be send to all participating patients by email. After completing the baseline questionnaire all patients will be randomized into one of the three study groups.

### Randomization procedure

After providing informed consent and baseline measurement, randomization will be performed by an independent person with the use of a computer generated randomization list using block randomization with random block sizes between 3 and 10. This sequence is kept hidden from all involved researchers. It can be expected, that patients referred by a GP and patients referred by a sports physician are different in terms of BMI and activity level. These have both been described as possible risk factors for plantar fasciopathy and therefore might influence treatment response [8]. For this reason the randomization will be stratified for type of referral (GP or sports physician). As a consequence patients from both populations will be equally distributed across the three treatment arms.

### Interventions

Patients (*n* = 47) randomized to the usual care group will receive an information booklet with general information on plantar fasciopathy. This information booklet will contain information on the use of pain medication and possible stretching and strength exercises (plantar specific stretching identical to the protocol used by DiGiovanni et al. [25] and unilateral heel raises adapted from exercises as described by Rathleff et al. [26]) that are considered to be helpful for all patients with plantar fasciopathy. The patients in this group will not be referred to a podiatrist. They will receive usual care as provided by their GP or sports physician.

Patients randomized to the custom made insole group (*n* = 69) and the placebo insole group (*n* = 69) will a be referred to a podiatrist. All patients that are referred to a podiatrist will additionally receive the information booklet identical to the usual care group as described above. The first appointment with the podiatrist will take place as soon as possible after randomization (preferably within 1 week). All patients referred to a podiatrist will receive a standardized intake with identical procedures. These procedures have been discussed and agreed upon with the participating podiatrists in a consensus meeting. If a podiatrist could not attend they were given elaborate explanation and the opportunity to give input during a telephone conference.

The standardized intake at the podiatrist will consist of an examination of the posture of the foot using the standardized Foot Posture Index [27], the Hubscher test [28], the navicular drop test [29] and the range of motion in the tarsometarsal joint and the first metatarsophalangeal joint (MTP-I) as measured with a goniometer. The podiatrists will report for each patient, whether they agree with the indication for orthotic insoles as established by the GP or sports physician. A 3D imprint of the feet of all patients visiting a podiatrist will be made. All these examinations will take place during the first appointment, which will take approximately 50 min. During this first appointment the podiatrist is blinded for the outcome of the randomization. The podiatrist will be informed about the allocated treatment, after the first appointment.

**Fig. 2:**
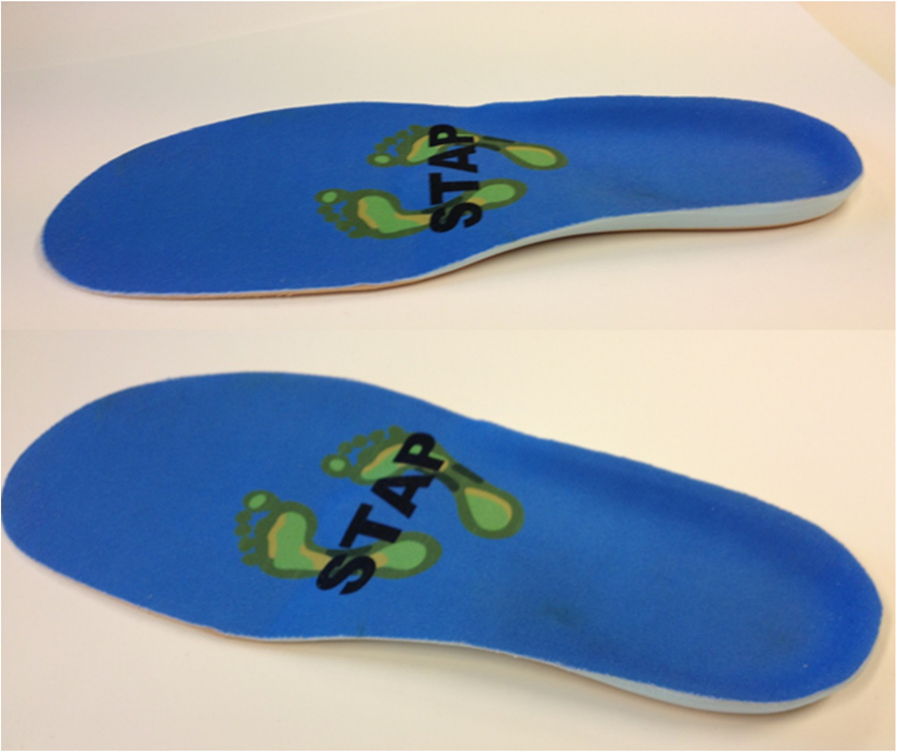
The placebo insole

**Fig. 3:**
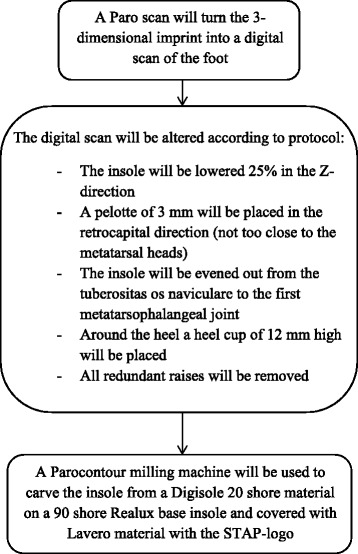
The procedure for producing the placebo insole

All patients referred to a podiatrist will have a second appointment within 2 weeks after the intake, of approximately 10 min to pick up their insole. Patients randomized to the custom made insole group will receive a custom made insole. Patients randomized to the placebo insole group will receive a placebo insole (Fig. 2), that has been custom designed for their foot (based on a 3D imprint of their foot) without providing therapeutic effect, but still have the visual effect of a podiatric insole. See Fig. 3 for the standardized production procedure of the placebo insole. All placebo insoles will be produced by the same podiatrist according to this protocol after receiving the 3D imprint from the different podiatrists. The placebo insoles will then be sent back to the different podiatrists to give to the patients. The custom made insole will always be patient tailored, but will be produced according to usual podiatric care by the different podiatrists. A third checkup appointment will be offered to the patients in the placebo insole group and the custom made insole group after 12 weeks as part of the usual podiatric care. Podiatrists with a practice near participating GP’s and sports physicians were approached to participate in the study. For every participating GP or sports physician a nearby podiatrist was found. In total 49 podiatrists have agreed to participate in this study.

### The use of co-intervention

In the 6 months of follow-up the GP’s and sports physicians will provide their usual treatment to the patients. They are discouraged from referring patients in the usual care group to the podiatrist during follow-up. Patients who receive co-interventions, will not count as a failure in the analysis. The podiatrists will be allowed to give advice on shoes and exercises as part of the podiatric intervention. Patients will report co-interventions in the questionnaires.

### Study outcomes

The primary outcome will be the change from baseline to 12 weeks follow-up in pain severity at rest and during activity on a 0–10 numerical rating scale (NRS). Secondary outcome measures include foot function as measured by the Foot Function Index at 6, 12 and 26 weeks follow-up, perceived recovery measured on a 7-point Likert scale (ranging from “worse than ever” to “completely recovered”, those who rate themselves as “slightly recovered” to “worse than ever” will be deemed not recovered) at 6, 12 and 26 weeks follow-up, pain at rest and during activity at 6 and 26 weeks follow-up and cost-effectiveness of the intervention at 26-weeks follow-up.

### Measurements

During a 6 month follow-up the participating patients will be asked to complete a total of six questionnaires. At baseline, at 2 weeks, at 4 weeks, at 6 weeks, at 12 weeks and at 26 weeks of follow-up. The participants will receive an e-mail that contains a secured hyperlink to the questionnaire, using the survey application Lime Survey. Phone calls and reminder emails will be used to minimize loss to follow-up and missing data.

### Baseline measurement

The baseline questionnaire will include questions on demographics (age, gender, BMI, education level, work and comorbidities), duration of pain, work activity (type, magnitude and load), sports activities (including the validated questionnaires SQUASH and Tegner) [30, 31], general health (SF12) [32], quality of life (EuroQol 5 dimensions) [33], neuropathic pain (DN4) [34], function (Foot Function Index (FFI)) [35], disability (Manchester-Foot Pain and Disability Index questionnaire (MPDI) [36], pain at rest and during activity (11-point NRS-scale) [37], incidence of falling, type of shoes and previously received medical care.

### Follow-up measurements

The 2, 4 and 6 week questionnaires will include questions on recovery on a 7-point Likert scale [38], pain at rest and during activity (NRS) and foot function (FFI). The questionnaires at 12 and 26 weeks will include these questions as well and will additionally include questions on general health (SF12), quality of life (EuroQol), direct cost (medical costs: health care visits, medication, medical devices), indirect cost (absence from work, inability to work) and current activity level. The patients randomized to the two podiatrist groups will also complete questions on patient satisfaction and compliance to treatment in the questionnaires at 12 and 26 weeks. In the 26 week questionnaire we will additionally ask all patients allocated to podiatric treatment to report their expectation regarding the type of insole they received, in order to assess the successfulness of blinding.

### Data analysis

Differences between the intervention groups will be analyzed following the intention-to-treat principle. Linear regression techniques for repeated measurements will be used to compare pain severity between the intervention group and control groups, which take the correlation of multiple measurements within one patient into account. A Bonferroni correction will be performed for the secondary comparisons to the usual care group. The analysis will be adjusted for potential confounders (at least including age, gender, BMI and activity level).

Recovery, pain scores and function will be analyzed using regression techniques for repeated measures using generalized mixed models.

Mean direct, indirect and total costs will be estimated and compared between the three study groups. Because costs will not be normally distributed, 95 % confidence intervals for the differences in mean costs will be obtained by bias corrected and accelerated bootstrapping (2000 replications). Differences in costs and differences in recovery will be included in a cost-effectiveness ratio, which estimates the additional costs to completely recover one patient.

Confidence intervals for the cost-effectiveness ratio will be calculated with bootstrapping, using the bias-corrected percentile method with 5000 replications. An incremental cost-effectiveness ratio will be estimated of the incremental costs to recover one patient. Uncertainty of this ratio will be evaluated by presenting a cost-effectiveness plane and sensitivity analyses will be performed to check the robustness of the results. An acceptability curve will also be presented.

Pre-defined subgroup analysis will be performed in patients with a long (>12 months) and short (<12 months) duration of the complaints at baseline, in patients with a low, moderate or high total activity score derived from the SQUASH questionnaire (in tertiles), in patients recruited in general practice and by sports physicians and in patients where the podiatrist agrees with the indication for orthotic insoles and in patients where the podiatrist does not.

## Discussion

In order to explore the effectiveness of custom-made insoles, we will compare the insole to a placebo insole. One earlier study found similar effects on pain and function at both short and long-term follow-up between placebo insoles and custom made podiatric insoles [18]. Since both groups showed improvement during follow-up, the improvements seen in pain reduction might be caused by either a real placebo effect or the natural course of the condition. Therefore, we will also compare custom made insoles with usual care.

It has also been acknowledged that the placebo effect plays a role in clinical trials using orthotic devices and that the development of a placebo intervention in these trials is a difficult task [39]. For the current study purpose we will design a custom placebo insole for each foot separately. As a consequence each subject in the placebo insole group will be provided with an insole that gives as little support as possible. The material and the features of the custom-made insole cannot be identical to the placebo insole, since this would interfere with its function. However, we will make the placebo insole look as similar to the orthotic insole as possible (in shape), by producing placebo insoles tailored for each patient. By doing this, the placebo insoles will have a 3D effect (but lower than the custom made insole), without expecting therapeutic effects. By making both type of insoles look as similar as possible, we hope to achieve successful blinding of patients for the type of treatment received. We will assess success of blinding of patients by means of a single question in the 26 week questionnaire.

We expect to find a larger difference between the custom made insole group and the usual care group, compared to the custom made and placebo insole groups, since patients allocated to the usual care group are likely to have a different attitude towards their treatment. It is worthwhile to study these differences since this provides information on the treatment with custom made podiatric insoles compared to other strategies used in daily practice and on the possible placebo effect involved with treatment with insoles. This knowledge will improve the insight into the treatment of plantar fasciopathy as it is currently given in clinical practice.

We will include patients from both general practice and sport medicine clinics. Previous studies have suggested possible subgroups of patients, which might be represented by the two different recruitment settings. For example, patients recruited in general practice are more likely to be middle-aged with perhaps higher mean BMI’s, whereas patients recruited by sports physicians are probably more physically active compared to those in general practice. By performing a stratified randomization for type of referral we will be able to analyze the possible difference in effectiveness of insoles in subgroup analysis.

Our sample size calculation is based on the data from Landorf et al. [18] This trial is to tour knowledge the only trial that has compared a custom made insole to a sham insole. The pain score in this trial was derived from the Foot Health Status Questionnaire, which is a pain score with a scale from 0 to 100. The primary outcome of the present study is pain severity measured on a 0–10 point NRS-scale. In order to determine our sample size, we have rescaled the pain severity outcomes for application on the 0–10 NRS.

The strength of this study is that we will perform a trial in which we will compare custom made insoles, a preferred treatment in patients with plantar fasciopathy, to a placebo treatment and usual care. The results will contribute to clinical decision making in primary health care when it comes to plantar fasciopathy and will additionally provide information on the cost-effectiveness of podiatric care including orthotic insoles.
